# MOMENT – Management of Otitis Media with Effusion in Cleft Palate: protocol for a systematic review of the literature and identification of a core outcome set using a Delphi survey

**DOI:** 10.1186/1745-6215-14-70

**Published:** 2013-03-12

**Authors:** Nicola L Harman, Iain A Bruce, Peter Callery, Stephanie Tierney, Mohammad Owaise Sharif, Kevin O’Brien, Paula R Williamson

**Affiliations:** 1 The Healing Foundation Cleft and Craniofacial Clinical Research Centre supported by VTCT, School of Dentistry, University of Manchester, L5.CT.364 (Research Floor), St Mary’s Hospital Oxford Road, Manchester M13 9WL, UK; 2 Central Manchester University Hospitals NHS Foundation Trust, Oxford Road, Manchester M13 9WL, UK; 3 School of Nursing, Midwifery & Social Work, University of Manchester, Jean McFarlane Building, Oxford Road, Manchester M13 9PL, UK; 4 Department of Biostatistics, University of Liverpool, Shelley’s Cottage, Brownlow Street, Liverpool L69 3GS, UK

**Keywords:** Core outcome set, Delphi, Consensus methods, Cleft palate, Otitis media with effusion

## Abstract

**Background:**

Cleft palate (CP) has an incidence of approximately 1 in 700. Children with CP are also susceptible to otitis media with effusion (OME), with approximately 90% experiencing nontrivial OME. There are several approaches to the management of OME in children with CP. The Management of Otitis Media with Effusion in Children with Cleft Palate (MOMENT) study is a feasibility study that includes the development of a core outcome set for use in future trials of the management of OME in children with CP.

**Methods/Design:**

The MOMENT study will include a systematic review of the literature to identify a list of outcomes that have previously been reported. This list of outcomes will be used in a Delphi study with cleft clinicians. The Delphi study is anticipated to include three rounds. The first round will ask clinicians to score the outcome list and to add any outcomes they think are relevant. The second round involves presentation of scores according to stakeholder group and the opportunity for participants to rescore outcomes. To ensure that the opinion of parents and children are sought, qualitative interviews will be completed with a purposive sample in parallel. In the final round of the Delphi process, participants will be shown the distribution of scores, for each outcome, for all stakeholder groups separately as well as a summary of the results concerning outcomes from the qualitative interviews with parents. A final consensus meeting will be held with all stakeholders, including parents and children, to review outcomes.

**Discussion:**

A core outcome set represents the minimum that should be measured in a clinical trial for a particular condition. The MOMENT study will aim to identify a core outcome set that can be used in future trials of the management of OME, improving the consistency of research in this clinical area.

## Background

Cleft lip and palate are among the most common congenital malformations, with an overall incidence of around 1 in 700 individuals [1]. Cleft palate (CP) results in impaired Eustachian tube function; children with this condition are therefore susceptible to otitis media with effusion (OME), and approximately 90% of children with CP have a history of nontrivial OME [1,2]. OME can impair hearing at stages thought to be important in the development of language, behavioural and social relationships. As a consequence it can influence the quality of life in these individuals.

There are several approaches to the management of OME in children with CP, including watchful waiting, the provision of hearing aids, the insertion of ventilation tubes (grommets) or a combination of these [3]. However, treatment protocols for OME have not always been well defined. Recently, National Institute for Clinical Excellence Guideline CG60 on the surgical management of OME in children recommended a general care pathway of watchful wait for 3 months followed by the insertion of ventilation tubes or the provision of hearing aids if necessary. In a systematic review directed at the early routine insertion of ventilation tubes for management of OME for children with CP, the authors identified 18 eligible studies but only one of these was a randomised control trial [4]. Overall the quality of the studies – determined by sample size and selection, performance and reporting bias – was low, with many being small and without sample-size calculations.

The approach to the treatment of OME in children with CP is further complicated by the different clinical stakeholder groups involved in the management of these patients and, although cleft care has been centralised in the UK to 12 hub centres, audiological and ear nose and throat treatment may involve visits to other hospitals outside the cleft centre.

The Management of Otitis Media with Effusion in Children with Cleft Palate (MOMENT) study has been funded through a Health Technology Assessment-commissioned call (project number 09/167) to address the uncertainty in the treatment of OME and to address the question ‘What is the most appropriate way to manage otitis media with effusion in children with cleft palate?’ by completing a feasibility study.

### Selection of outcomes for use in clinical trials of treatment for OME in children with cleft palate

Clinical trials should have defined primary and secondary outcomes that answer questions generated by the main hypotheses. However, when we consider outcomes that may be used in studies of the treatment of OME in children with CP, it appears that these are numerous and diverse, and include outcomes such as chronic otitis media, OME, hearing loss, Eustachian tube function, behaviour, receptive language and side effects of treatment, to name a few. Furthermore, these may be influenced by other factors associated with clefting; for example, the effect of the palatal cleft on speech.

This diversity has been illustrated by a recent review that aimed to identify core outcome sets (COSs) for trials of treatment of childhood conditions. The authors categorised outcomes measured in clinical trials for a variety of paediatric conditions into six broad domains: disease activity, physical consequence of disease, functional status, social outcome and quality of life, side effects of therapy, and health resource utilisation [5]. Importantly, in this review they did not retrieve any studies ascertaining agreed core outcome domains and endpoints for OME.

In summary, it appears that the domains with the most tangible benefits for the users and providers of care for children with CP and OME are unknown, and traditionally researchers have used diverse outcomes. This situation could lead to the following potential problems.

#### Heterogeneity between studies

This heterogeneity may be illustrated by considering the findings of a recently published systematic review directed at the early routine insertion of ventilation tubes for management of OME in children with CP. In this review the authors evaluated the literature up to 2006 (including randomised controlled trials, controlled clinical trials, case series and historical cohort studies) [4]. Eighteen studies satisfied the inclusion criteria. When the outcomes were evaluated, the studies were shown to have used varied primary outcome measures including: hearing loss; tympanosclerosis; parental satisfaction with treatment; speech and language; and OME. Furthermore, there was inconsistency in the method of assessment for some outcomes in particular speech and language, which was assessed by undefined speech and language therapist assessment, a study-specific scale or using the Reynell Developmental Language Score. This limited consistency between studies, leading to marked heterogeneity, may result in difficult interpretation and comparison of findings and may hinder potential meta-analysis [5,6].

#### Outcome reporting bias

Another relevant factor is outcome reporting bias. This bias occurs when only a selection of results for measured outcomes is reported in a study. For example, a tendency to report only significant or positive findings results in a biased representation of the results of a trial. There is overwhelming empirical evidence that this phenomenon occurs [7].

### Core outcome sets

One strategy that has been suggested to overcome these issues is the development of a COS, which should be measured and reported in all randomised control trials of a specific condition [5,6,8-11]. As a result, the risk of outcome reporting bias and heterogeneity is reduced and the potential for carrying out a meta-analysis for key outcomes is increased.

The outcome measures that could potentially be used to evaluate OME treatment are numerous and diverse, and may also be affected by specific factors in clefting. There is currently no COS available for clinical trials of the management of OME in children with CP.

### Aims and objectives

#### Aim

The aim of this study is to contribute to the development of a COS, relevant to the surgical treatment of OME suitable for use in studies in children with CP.

#### Objectives

The specific study objectives are: to identify a list of outcomes previously reported in studies of the treatment of OME from a systematic review of the literature; to prioritise outcomes from the clinician perspective; to prioritise outcomes from the perspective of patients who can express their views; to compare clinician important outcomes and patient/parent important outcomes; and to integrate patient/parent and clinician outcomes into a combined COS.

## Methods/Design

### Systematic review

The systematic review will be carried out using two sources. We will identify outcomes reported in studies of the early placement of ventilation tubes for children with CP by updating the search from a previous systematic review [4]. In addition, we will review outcomes measured in studies of other surgical interventions for OME in children with and without cleft by reviewing relevant Cochrane Reviews. This is an efficient approach that should provide generalisable results.

### Criteria for considering studies for updating the reviews

The criteria for considering studies for updating the Bristol review [4] and Cochrane Reviews identified through a Cochrane Central Register of Controlled Trials (CENTRAL) search are as follows.

#### Types of studies

Studies will include systematic reviews with/without meta-analyses, randomised controlled trials, case controlled trials, case series, and prospective cohorts.

#### Types of intervention

Any surgical intervention used to manage OME in children with and without CP.

#### Types of participants

Children aged <18 years with OME.

#### Exclusion criteria

Nonsurgical interventions for the treatment of OME.

### Search methods for identification of studies

We will use an identical search strategy to Ponduri and colleagues [4]. This search will be applied to the CENTRAL, EMBASE, MEDLINE, and the Cumulative Index to Nursing & Allied Health Literature (CINAHL) (January 2006 to present).

For the detailed search strategies applied to each of the databases, see Additional file 1.

Multiple databases will be utilised to maximise the sensitivity of a search. CENTRAL comprises only studies that are deemed to be controlled trials by a team of reviewers, EMBASE, MEDLINE and CINAHL include published research of various study designs. The advantages conferred by using CENTRAL in addition to the other databases are that trials from other sources of research (for example, journals not indexed in MEDLINE and conference proceedings) are hand searched, and controlled trials from these are included. This improves the chances of identifying all relevant studies.

#### Eligibility of studies

Two review authors (MOS and KOB) will independently assess the abstracts of studies resulting from the searches. Full copies of all potentially relevant studies and those appearing to meet the inclusion criteria, or for which there were insufficient data in the title and abstract to make a clear decision, will then be obtained.

The full-text papers will be assessed independently by two review authors (MOS and KOB) and any disagreement on the eligibility of included studies resolved through discussion. Where resolution is not possible, a third review author (IAB) will be consulted.

#### Assessment of methodological quality

The quality of describing and reporting the outcomes will be assessed within each study by considering the following questions:

1. Is the primary outcome clearly stated?

2. Is the primary outcome clearly defined so that another researcher would be able to reproduce its measurement? Where appropriate, this should include clear description of time points, the person measuring the outcome, how the outcome was measured (for example, tools and methods used) and where the outcome was measured.

3. Are the secondary outcomes clearly stated?

4. Are the secondary outcomes clearly defined?

5. Do the authors explain the use of the outcomes they have selected?

6. Are methods used to enhance the quality of outcome measurement (for example, repeated measurement, training) if appropriate?

For the purpose of this study there will be no synthesis of outcome data from the randomised control trials, and hence a critique of the overall methodological quality of the studies themselves is not necessary.

#### Data extraction

In the first instance, data will be extracted independently and in duplicate by two review authors (NLH and IAB). NLH and IAB will then review the extracted data together to assess consensus and to ensure that all outcomes have been identified. Disagreement will be resolved through discussion; where resolution is not possible, a third review author (PRW/KOB) will be consulted. In addition, we will contact study authors in order to identify unavailable/unclear data.

The following data will be extracted from each study: study type; author details; year and journal of publication; intervention(s) under investigation; whether the study population was exclusively paediatric and CP or mixed (children and adults, with or without CP); age and number of children included in the study population; and inclusion and exclusion criteria.

#### Outcomes

The MOMENT study outcomes will include: the outcomes that were measured, including the method of measurement; the time points at which they were measured; if stated, the designated primary outcome; and the designated secondary outcome(s).

#### Data analysis and presentation

For analysis purposes, the data will be initially tabulated so that each study is listed, and the outcomes measured in the trial are displayed.

Outcomes will be grouped under appropriate outcome domains. The outcome domains will be determined following a review of the extracted outcomes by the authors (IAB and NLH). The outcome domains and included outcomes will be reviewed by the Study Advisory Group to assess suitability of the domain name and grouping of outcomes.

Within each domain we will be able to evaluate both how many different outcomes have been used to reflect that domain and the frequency of selection for each individual outcome, and the times at which they were measured will be documented.

### Identification of outcomes of importance to parents and patients

The opinions of parents and children on the treatment of OME for children with CP are important because it is this group who will experience the benefits and adverse effects of treatments and they should have opportunities to contribute to identification of the most appropriate outcomes. The identification of outcome domains important to children and parents will be grounded in their own accounts of what matters to them about OME and its treatment, in keeping with the principle of privileging the lay perspective [12].

A purposive sample of children aged 0 to 11 years with nonsyndromic CP and experience of either surgery for insertion of ventilation tubes or conservative management with hearing aids will be identified from two cleft lip and palate centres. Parents or carers, and, where able and willing, children aged at least 6 years will be interviewed. Qualitative sample sizes are estimated pragmatically in order to achieve the objective of saturation. Saturation means that the widest range of views is represented in the data. A sample of 30 is estimated on the basis of past experience to be sufficient for saturation, but the sample size will be increased as resources allow if new opinions are continuing to emerge in the final interviews.

The purposive approach to sampling will be used to include a diversity of age group, gender and treatment types. The aim will be to recruit in accordance with the purposive sampling matrix (Table 1). However, the final composition of the sample will depend on treatment practices in each age group. We will seek to maximise the representation of ethnic and demographic diversity of the populations in the recruitment centres in the sample.

**Table 1:** Purposive sampling matrix of parents and children

**Child age (years)**	**Treatment**				**Total**
	**Ventilation tubes**	**Hearing aids**	**Ventilation tubes and hearing aids**	**Watchful waiting**	
Parent sampling matrix					
0 to 5	3	3	2	2	10
6 to 8	3	3	2	2	10
9 to 11	3	3	2	2	10
Total	9	9	6	6	30
Child sampling matrix					
6 to 8	3	3	2	2	10
9 to 11	3	3	2	2	10
Total	6	6	4	4	20

Participants will be identified by cleft teams at participant centres to ensure that they are eligible for participation. Eligibility is based on a history of OME, an absence of a known syndrome and the ability to complete the interview in English.

The qualitative interviews will take the form of conversations in which parents tell the stories of their child’s cleft, OME and its treatment, and the consequences for the child and their families. This open-ended approach will enable parents to raise issues of most importance to themselves and the interviewer to explore parents’ understanding of alternative treatments. A topic guide will be used to ensure that all interviews address a set of core issues, including important outcomes of treatment. Interviews will also be completed with children aged at least 6 years and interviews adapted to meet their developmental needs. Children who have lived with cleft and OME may have views about the way their condition is treated and should be consulted because it is they who have the most direct experience treatment outcomes. Visual techniques will be employed, including drawing pictures and using interactive screen tablet computer applications.

All interviews will be recorded and transcribed. Recordings of consultations will be imported into qualitative analysis software and analysed using the five stages of the framework approach: familiarisation with the data (becoming immersed in material collected); development of a thematic framework (identifying key issues in the transcripts); indexing data (labelling key issues that emerge across cases); devising a series of thematic charts (allowing the full pattern across cases to be explored and reviewed); and mapping and interpreting data (looking for associations, providing explanations, highlighting key characteristics and ideas) [13]. This will provide a summary of participants’ key points about what they consider important and their priorities. Interview data that take the form of narrative explanations of the effect of OME and treatments on participants’ lives will be interpreted by the process of constant comparison [14] to identify outcome domains and to understand their importance to children and parents and their rationale for their priorities. These outcome domains will be identified independently to the outcomes identified from systematic review of the literature.

Ethical approval for the qualitative interviews with parents and children has been sought and granted by the National Research Ethics Service – NRES North East Committee – Greater Manchester East (reference 11/NW/0586).

### Identification of outcomes of importance to clinicians

#### Overview

To investigate outcomes of importance to clinicians, a Delphi approach has been adopted so that the anonymous opinions of the clinicians can be obtained in a way that gives equal influence to all who participate, and avoids an individual participant being overtly influenced by the opinions of any other participant [15,16]. An overview of the Delphi exercise is given in Additional file 2.

#### Identification of potential outcomes

An initial list of potential outcomes for use in the Delphi exercise will be obtained from the systematic review described above. To aid interpretation of the outcomes by clinicians and to improve the ease of use of the Delphi system, all outcomes will be listed individually but also grouped under a relevant domain.

Following initial review of the outcome list by the MOMENT Study Management Group Clinician (IAB), the list will be circulated to the MOMENT Study Advisory Group (SAG).The SAG is comprised of cleft clinicians representing speech and language therapists, cleft surgeons, ear nose and throat surgeons, audiologists and clinical psychologists.

Members of the SAG will be asked to review outcomes relevant to their clinical field. The SAG will be asked to review the list of outcomes for comprehension and will also be asked to comment on the suitability of the overall domain under which outcomes are grouped.

The members of the study advisory group will also be asked to participate in their own round 1 of the Delphi exercise and to list any additional outcomes that they think should be included. Additional outcomes identified by the SAG will be coded and added to the list of outcomes used in the first round of the Delphi exercise that is sent to all clinicians. This review process will produce a final list of outcomes identified from both the systematic review and additional outcomes suggested by the SAG. Owing to their involvement in the study design and contribution to the Delphi exercise as an expert panel, discussions between stakeholder groups within the SAG may influence the scoring of outcomes. For this reason, members of the SAG will not be invited to participate in the Delphi exercise. Instead, members of the SAG will be invited to participate in the final consensus meeting and to chair relevant stakeholder sessions if appropriate.

Outcomes identified as patient-reported outcome measures or other validated tools will be reviewed and the domains used within the tool used as an outcome instead of the tool itself. Where there is uncertainty about how to present these outcomes, the advice of two relevant clinicians who are members of the SAG will be sought.

Outcomes identified from the systematic review that are related to resource use will not be included in the outcomes list used in the Delphi exercise. Information on resource use that is needed for a future trial will be identified using modelling and value of information analysis.

#### Participants

The Delphi study will be conducted with the clinical teams from all UK cleft centres. Clinicians will be selected only from UK centres due to time and cost constraints. Key clinicians are audiologists, ear nose and throat surgeons, speech and language therapists and specialist nurses. Other clinicians – for example, paediatricians, clinical psychologists, clinical geneticists and cleft surgeons – will be identified after consultation with the clinical director or cleft service coordinator at each cleft centre. Clinicians will only be invited to participate if they are involved in the clinical care of OME in children with CP.

Identified clinical leads will be approached by email and asked to provide contact details for all clinicians who are part of the cleft team at their centre. Individual participants will then be emailed directly and asked to complete an online Delphi questionnaire via an embedded link.

The number of participants invited to participate will be documented and recruitment to round 1 assessed. The number of participants completing subsequent rounds will also be documented and attrition assessed.

At the beginning of the exercise, participants will be reminded of the importance of completing the entire Delphi process. Reminder emails will also be sent to aid completion of each round. Participants who agree to take part will be asked to register and a unique identifier will be allocated to enable tracking of attrition at each round. Upon registration, participants will be asked to specify their clinical role to allow identification of stakeholder groups. All data will be stored against the unique identifier only; participants will not be able to identify other participants or individual responses.

All members of cleft teams will be eligible to participate and do not need to have had prior involvement in clinical trials. Although all team members identified will be invited to participate, it is expected that some clinical roles will not complete the survey due to a lack of expertise in the treatment of OME. These roles may include orthodontists and paediatric dentists. Specification of clinical role as part of the registration process will identify minority clinical groups and the presentation of results in round 2 determined following an analysis of the numbers of participants in each stakeholder group.

The National Research Ethics Committee has been consulted and confirmed that the Delphi survey with clinicians does not require ethical approval.

### Delphi survey

#### Delphi round 1

In the first round the online questionnaire will request the participant’s name and email address together with their cleft centre. This information will be stored in a separate database and used to provide the respondent with a unique identifier. A unique identifier will allow identification of individuals completing all rounds of the Delphi exercise.

Each respondent will be asked to identify their clinical role (that is, audiologist, speech therapist, and so forth) in the first survey question. Participants will also be asked for their experience in clinical research.

Participants will be asked to complete each round of the Delphi exercise within 3 weeks of receipt of the email and will be reminded of this at the start of each survey. A reminder email will be sent at the end of week 2 to prompt completion of the survey.

#### Round 1 survey format

The survey will be presented in an online format (see Additional files 3 and 4).

Round 1 content includes: the respondent’s clinical role; a list of outcomes to be scored, ordered alphabetically; and an option for a participant to add any additional outcomes and to provide a score for each outcome added.

At the beginning of the survey, participants will be presented with the information detailed in Additional file 3. They will be asked the key question: ‘What outcomes influence your management of children with cleft palate, with, or at high risk of, otitis media with effusion (OME)?’

Participants will be asked to score each of the outcomes listed using the Grading of Recommendations, Assessment, Development and Evaluations scale of 1 to 9. In the Delphi exercise the scale will be presented in the format 1 to 9, with 1to 3 labelled ‘not important’, 4 to 6 labelled ‘important but not critical’ and 7 to 9 labelled ‘critical’ [17]. Participants will be provided with an option to add additional outcomes that they think are relevant together with a score for each outcome added.

Outcomes will be listed alphabetically to avoid potential weighting of outcomes caused by the order in which they are displayed.

### Analysis of round 1

Additional outcomes listed by participants will be reviewed and coded by two members of the study team (NLH and IAB) to ensure they represent new outcomes. If there is uncertainty then the Study Management Group will be consulted, and the SAG as appropriate. For each outcome, the number of participants who have scored the outcome and the distribution of scores (as percentage who have scored each outcome) will be summarised by stakeholder group. All outcomes will be carried forward to round 2.

### Response rate in round 1

The number of participants in each stakeholder group who respond to round 1 will be assessed following round 1 closure. Results will be presented as: the total number of registrations; a breakdown of respondents who have completed the survey and their inclusion in the initial email invitation; the total number of respondents who completed the round; the total number of respondents in each stakeholder group; the percentage of respondents compared with potential respondents as identified from the information provided by clinical leads; and the percentage of respondents from other sources (not included in original email invitation).

Continuation to round 2 will be considered based on the response to round 1. If a low number of responders (<10) is observed for one or more stakeholder groups, the Delphi protocol for future rounds will be reviewed and revised. Where there is only one stakeholder group with a small number of respondents (potentially due to the sample available from clinical teams) then consideration will be given to grouping with another stakeholder group. This will be done in consultation with the SAG to ensure appropriateness of grouping.

The following proposed approach assumes sufficient numbers of stakeholders from each group respond.

### Delphi round 2

Round 2 will be presented online. In round 2, each participant will be presented with the number of respondents and distribution of scores for each outcome for their particular stakeholder group. Participants will be shown their score from round 1, asked to consider responses from the other members of their stakeholder group, and asked to rescore the outcome. Participants will also be asked whether the outcome should be included in a COS.

In round 2, participants are asked to review the score provided in round 1 for each of the outcomes. Any changes to scores in light of the stakeholder group or overall response will be documented. Those who have not taken part in round 1 and have not provided a score will not be invited to participate in round 2.

### Analysis of round 2

The total number of participants invited to take part in round 2 will be recorded. For each outcome, the number of participants who have scored the outcome and the distribution of scores will be summarised by stakeholder group. All outcomes will be carried forward to round 3.

### Delphi round 3

In round 3, participants will be shown the distribution of scores, for each outcome, for all stakeholder groups separately as well as a summary of the results concerning outcomes from the qualitative interviews with parents. Participants will then be asked to rescore all outcomes and state whether they should be included in a COS. Round 3 will be presented online.

### Analysis round 3

The total number of participants invited to take part in round 3 will be recorded. For each outcome, the number of participants who have scored the outcome and the distribution of scores will be summarised together with the number of participants who have scored the outcome in all rounds. Results of the stakeholder group response will be compared with the whole group response and the percentage agreement used to determine the structure and focus of the final consensus meeting. Each outcome will be classified as ‘consensus in’, ‘consensus out’ or ‘no consensus’ according to the classifications in Table 2.

**Table 2:** Definitions of consensus

**Consensus classification**	**Description**	**Definition**
Consensus in	Consensus that outcome should be included in the core outcome set	70% or more participants scoring as 7 to 9 AND <15% participants scoring as 1 to 3
Consensus out	Consensus that outcome should not be included in the core outcomes set	70% or more participants scoring as 1 to 3 AND <15% of participants scoring as 7 to 9
No consensus	Uncertainty about importance of outcome	Anything else

### Consensus meeting

The final phase of the study will be a face-to-face consensus meeting with all participants of the clinician Delphi exercise and the qualitative study invited. Members of the SAG will be invited to chair relevant stakeholder sessions as appropriate.

The results from each round of the Delphi survey will be presented. Review of the responses from clinicians in round 3 of the Delphi exercise and interviews with parents and children will be used to inform the structure and content of the consensus meeting.

Prior to the consensus meeting, participants of the qualitative interviews will also be given the opportunity to view the responses in the clinician Delphi exercise.

The final format of the consensus meeting will be determined following review of the experiences of previous similar projects and the agreement between the total group scores and stakeholder group scores.

### Definition of consensus

The classification described in Table 2 will be used when determining whether consensus is reached or not.

To have reached consensus that an outcome should be in the COS requires agreement by the vast majority regarding the critical importance of the outcome, with only a small minority considering it to be not important at all. Conversely, for consensus to have been reached that an outcome should not be in the COS requires agreement by the vast majority regarding the lack of importance of the outcome, with only a small minority considering it to be critically important. Whilst the choice of thresholds is inevitably somewhat subjective, this specification of the definition of consensus upfront should reduce the chance of consensus being defined *post hoc* in such a way as to bias the results towards the beliefs of the research team.

### Statistical considerations

#### Sample size

There is currently no standard method for sample size calculation in Delphi processes, and thus a pragmatic approach is taken. The following sample sizes are expected to yield a meaningful statistical analysis. However, the number of participants in the present study is limited by the composition and number of UK cleft centres. Efforts will be taken to maximise the response rate across centres and stakeholder groups.

For this study, all key clinicians from all 16 UK cleft centres (which comprise 12 UK cleft services) will be invited to take part. The total number of respondents in each stakeholder group will be reviewed after round 1. Based on the information currently available for eight of the cleft centres, the estimated sample sizes for all 16 centres by clinician stakeholder group (adjusted for an anticipated 70% response rate) are as follows: audiologist, *n* = 16 (adjusted *n* = 11); cleft surgeon, *n* = 44 (adjusted *n* = 31); ear nose and throat surgeon, *n* = 30 (adjusted *n* = 21); paediatrician, *n* = 12 (adjusted *n* = 8); specialist cleft nurse, *n* = 52 (adjusted *n* = 36); speech and language therapist, *n* = 88 (adjusted *n* = 62); and psychologist, *n* = 24 (adjusted *n* = 17).

## Discussion

There is currently no published COS for OME. The development of a COS in this clinical area aims to improve the interpretation and comparison of future studies and to reduce the risk of outcome reporting bias and heterogeneity across studies. The MOMENT study will involve multiple key stakeholder groups to ensure that a COS is suitable and well accepted in future research.

## Abbreviations

CENTRAL: Cochrane Central Register of Controlled Trials; CINAHL: Cumulative Index to Nursing & Allied Health Literature; CP: cleft palate; COS: core outcome set; MOMENT: Management of Otitis Media with Effusion in Children with Cleft Palate; OME: otitis media with effusion; SAG: Study Advisory Group.

## Competing interests

The authors declare that they have no competing interests.

## Authors’ contributions

PRW and KOB conceived of the study, and participated in its design and helped to draft the manuscript. PRW, KOB and MOS designed the protocol for the systematic review. MOS and KOB completed the systematic review search and extraction of eligible papers. NLH and IAB extracted outcomes for the outcomes list and drafted the list of outcomes for the online Delphi. NLH and PRW developed the Delphi protocol. NLH wrote the first draft the manuscript. ST, MOS and PC have designed the protocol for qualitative interviews. ST completed qualitative interviews with parents and children. All authors edited the manuscript and read and approved the final version.

## Supplementary Material

Additional file 1 Systematic review search strategies. Click here for file

Additional file 2 Overview of Delphi process. Click here for file

Additional file 3 Delphi survey formats. Click here for file

Additional file 4 Online system screenshots. Click here for file
