# Supplementary material for: MOMENT – Management of Otitis Media with Effusion in Cleft Palate: protocol for a systematic review of the literature and identification of a core outcome set using a Delphi survey
Source: Trials. 2013 Mar 12;14:70. doi: 10.1186/1745-6215-14-70 (PMC3716725; doi:10.1186/1745-6215-14-70)
Supplement: Additional file 1 — Systematic review search strategies. [file 1745-6215-14-70-S1.pdf]

Additional File 1. Systematic review search strategies.

CENTRAL via OVID search strategy

2006-14/04/2011

#1 MeSH descriptor cleft palate explode all trees 92
#2 MeSH descriptor cleft lip explode all trees 69
#3 (cleft* in All Text near/6 palate* in All Text) 176
#4 (cleft* in All Text near/6 lip* in All Text) 131
#5 "hare lip*" in All Text 2
#6 harelip* in All Text 2
#7 Palatoschisis in All Text 1
#8 (orofacial* in All Text near/6 cleft* in All Text) 6
#9 (facial* in All Text near/6 cleft* in All Text) 13
#10 (face* in All Text near/6 cleft* in All Text) 7
#11 (#1 or #2 or #3 or #4 or #5 or #6 or #7 or #8 or #9 or #10) 204
#12 MeSH descriptor middle ear ventilation explode all trees 200
#13 MeSH descriptor otitis media explode all trees 820
#14 grommet* in All Text 72
#15 (ear in All Text near/6 ventilat* in All Text) 232
#16 otitis next media in All Text 1527
#17 (ventilat* in All Text near/6 tube* in All Text) 408
#18 tympanostom* in All Text 135
#19 (glue in All Text near/6 ear* in All Text) 65
#20 (#12 or #13 or #14 or #15 or #16 or #17 or #18 or #19) 1908
#21 (#11 and #20) 17

CINAHL search strategy

Via OVID (1982 to February Week 2 2006)

1 Cleft Palate/ (686)
2 Cleft Lip/ (526)
3 (cleft$ adj3 lip$).tw. (495)
4 (cleft$ adj3 palat$).tw. (617)
5 hare lip$.tw. (1)
6 harelip$.tw. (0)
7 Palatoschisis.tw. (0)
8 (orofacial$ adj3 cleft$).tw. (29)
9 (facial adj3 cleft$).tw. (40)
10 (oral adj3 cleft$).tw. (49)
11 (craniofacial adj3 cleft$).tw. (47)
12 or/1-11 (817)
13 Middle Ear Ventilation/ (224)
14 exp Otitis Media/ (1259)
15 grommet$.tw. (21)
16 (ear$ adj3 ventilat$).tw. (80)
17 otitis media.tw. (898)
18 (ventilat$ adj3 tube$).tw. (99)
19 tympanostom$.tw. (90)

20 (glue adj3 ear$).tw. (32)
21 or/13-20 (1634)
22 21 and 12 (17)
23 from 22 keep 1-17 (17)

Via EBSCO (February Week 2 2006 -13/04/2011)

	Cinahl Ovid				Cinahl Ebsco			
	Keyword	Field	Results		Keyword	Field	Results 1982 to current	Results for 1982-Feb 2006
1	Cleft Palate	Thesaurus	686		Cleft Palate	Thesaurus	1436	751
2	Cleft Lip	Thesaurus	526		Cleft Lip	Thesaurus	1099	567
3	cleft$ adj3 lip$	Text Word (.tw.)	495		Cleft* N3 lip*	TI, AB,IN	995	530
4	cleft$ adj3 palat$	Text Word (.tw.)	617		Cleft* N3 palat*	TI, AB,IN	1232	663
5	hare lip$	Text Word (.tw.)	1		hare lip*	TI, AB,IN	1	1
6	harelip$	Text Word (.tw.)	0		Harelip*	TI, AB,IN	0	0
7	Palatoschisis	Text Word (.tw.)	0		Palatoschisis	TI, AB,IN	1	1
8	orofacial$ adj3 cleft$	Text Word (.tw.)	29		Orofacial* N3 cleft*	TI, AB,IN	93	33
9	facial adj3 cleft$	Text Word (.tw.)	40		facial N3 cleft*	TI, AB,IN	69	40
10	oral adj3 cleft$	Text Word (.tw.)	49		oral N3 cleft*	TI, AB,IN	115	50
11	craniofacial adj3 cleft$	Text Word (.tw.)	47		craniofacial N3 cleft*	TI, AB,IN	74	45
12	or/1-11		817		or/1-11		1741	889
13	Middle Ear Ventilation	Thesaurus	224		Middle Ear Ventilation	Thesaurus	410	264
14	exp Otitis Media	Thesaurus	1259		exp Otitis Media	Thesaurus	2403	1467
15	grommet$	Text Word (.tw.)	21		grommet*	TI, AB,IN	49	28
16	ear$ adj3 ventilat$	Text Word (.tw.)	80		ear* N3 ventilat*	TI, AB,IN	158	75
17	otitis media	Text Word (.tw.)	898		otitis media	TI, AB,IN	1646	1104
18	ventilat$ adj3 tube$	Text Word (.tw.)	99		ventilat* N3 tube*	TI, AB,IN	266	166
19	tympanostom$	Text Word (.tw.)	90		tympanostom*	TI, AB,IN	171	110
20	glue adj3 ear$	Text Word (.tw.)	32		glue N3 ear*	TI, AB,IN	42	33
21	or/13-20		1634		or/13-20		3210	
22	21 and 12		17		21 and 12		36	
23	from 22 keep 1-17		17		from 22 keep 1-17			

EMBASE via OVID Search strategy

2006-14/04/2011

1 cleft lip/ (3181)
2 Cleft Palate/ (5513)
3 Cleft Lip Face Palate/ (257)
4 Cleft Lip Palate/ (931)
5 (cleft$ adj3 lip$).tw. (4006)
6 (cleft$ adj3 palat$).tw. (5885)
7 hare lip$.tw. (23)
8 harelip$.tw. (31)
9 Palatoschisis.tw. (25)
10 (orofacial$ adj3 cleft$).tw. (243)
11 (facial adj3 cleft$).tw. (675)
12 (oral adj3 cleft$).tw. (290)
13 (craniofacial adj3 cleft$).tw. (259)
14 or/1-13 (9179)
15 middle ear ventilation/ (238)
16 exp Otitis Media/ (13078)
17 tympanostomy tube/ (1111)
18 grommet$.tw. (305)
19 (ear$ adj3 ventilat$).tw. (901)
20 otitis media.tw. (9424)
21 (ventilat$ adj3 tube$).tw. (1075)
22 tympanostom$.tw. (640)
23 (glue adj3 ear$).tw. (216)
24 or/15-23 (16372)
25 14 and 24 (206)
26 from 25 keep 1-206 (206)

Medline via OVID search strategy

2006-14/04/2011

1 Cleft Palate/ (11628)
2 Cleft Lip/ (8131)
3 (cleft$ adj3 lip$).tw. (6156)
4 (cleft$ adj3 palat$).tw. (8901)
5 hare lip$.tw. (68)
6 harelip$.tw. (99)
7 Palatoschisis.tw. (69)
8 (orofacial$ adj3 cleft$).tw. (280)
9 (facial adj3 cleft$).tw. (759)
10 (oral adj3 cleft$).tw. (326)
11 (craniofacial adj3 cleft$).tw. (213)
12 or/1-11 (15658)

13 Middle Ear Ventilation/ (1469)
14 exp Otitis Media/ (15239)
15 grommet$.tw. (336)
16 (ear$ adj3 ventilat$).tw. (616)
17 otitis media.tw. (10785)
18 (ventilat$ adj3 tube$).tw. (1163)
19 tympanostom$.tw. (651)
20 (glue adj3 ear$).tw. (273)
21 or/13-20 (18849)
22 21 and 12 (297)
23 exp animals/ not human/ (2948249)
24 22 not 23 (286)
25 22 not 24 (11)
26 from 25 keep 1-11 (11)
