# Supplementary figures and images for: MOMENT – Management of Otitis Media with Effusion in Cleft Palate: protocol for a systematic review of the literature and identification of a core outcome set using a Delphi survey
Source: Trials. 2013 Mar 12;14:70. doi: 10.1186/1745-6215-14-70 (PMC3716725; doi:10.1186/1745-6215-14-70)

## Additional File 2 Overview of Delphi process

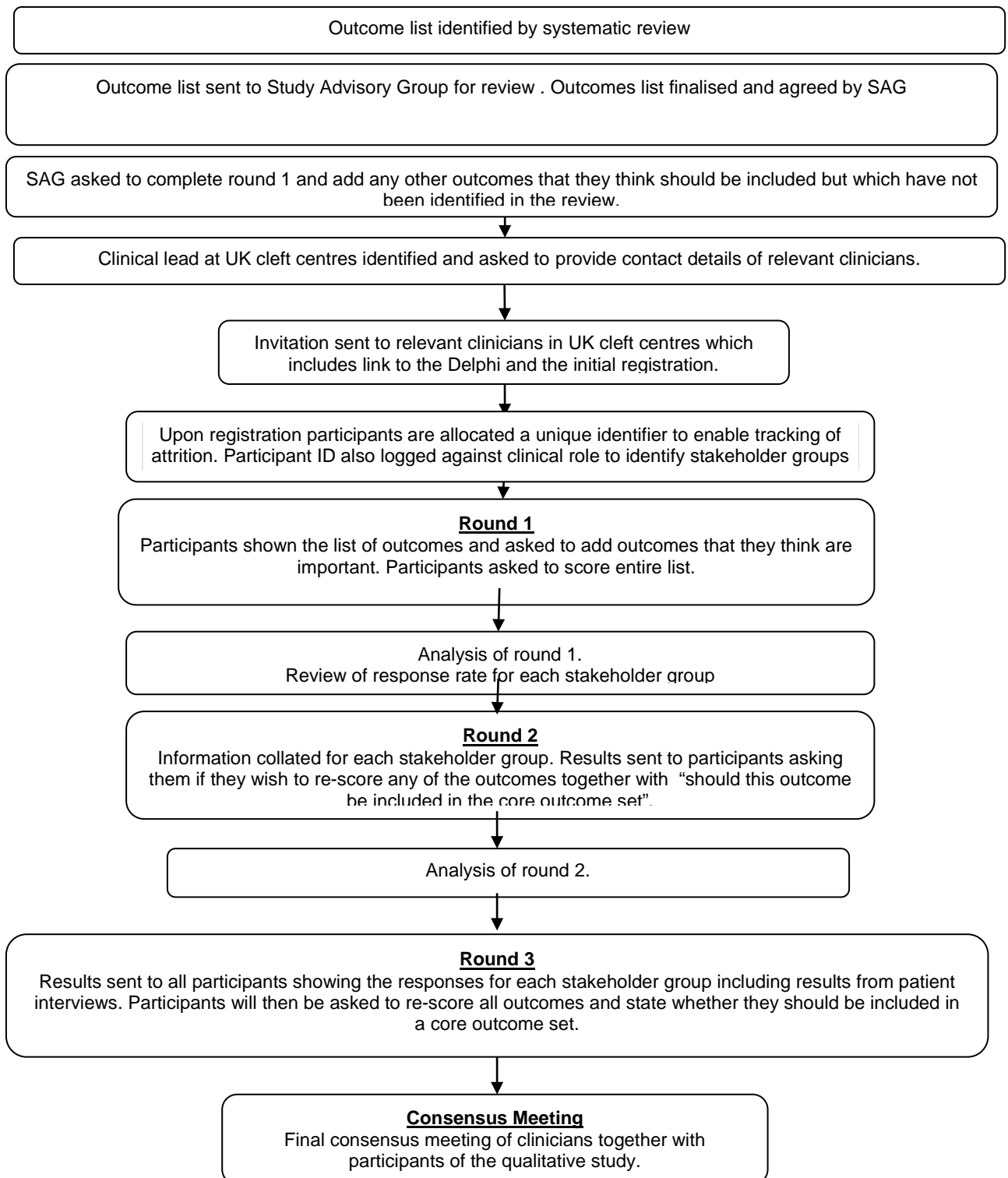

Supplement: Additional file 2 — Overview of Delphi process. [file 1745-6215-14-70-S2.pdf]
