# Supplementary material for: MOMENT – Management of Otitis Media with Effusion in Cleft Palate: protocol for a systematic review of the literature and identification of a core outcome set using a Delphi survey
Source: Trials. 2013 Mar 12;14:70. doi: 10.1186/1745-6215-14-70 (PMC3716725; doi:10.1186/1745-6215-14-70)
Supplement: Additional file 3 — Delphi survey formats. [file 1745-6215-14-70-S3.pdf]

Additional File 3 – Delphi Survey Formats

Surveys will be provided using a bespoke website and a unique link generated.

Phase one survey format
The survey will be presented in an online format and will be preceded by participant information.

Example:

MOMENT: Developing a core outcome set for children with cleft palate and otitis media with effusion.

Dear <first name>

Thank you for your interest in our study.

Core outcome sets represent the minimum that should be measured and reported in all clinical trials of a specific condition. Currently there is no core outcome set for studies of OME in children with cleft palate which may hinder the comparison of the effectiveness of interventions across trials.

The aim of this study is to find out which outcomes are important to clinicians and which outcomes should be included in a core outcome set for use in future trials in children with cleft palate and OME.

This questionnaire is the first round of three rounds of the survey.

We would like you to complete this questionnaire within three weeks (by the dd-mm-yy).

Once we have received responses from all panellists we will collate and summarise the findings and prepare the second questionnaire. Your responses in each round will be anonymous.

It is very important that you complete the questionnaires in each round. The reliability of the results could be compromised if people drop out of the study before it is completed, because they feel that the rest of the group does not share their opinions. If people drop out because they feel their opinions are in the minority, the final results will overestimate how much the sample of participants agreed on this topic.

All responses will be anonymous. However, to help us track completion of each round we would like you to initially register using your email address. Upon registration you will be allocated a unique identifier which will be used to process all data anonymously.

To access the survey please click on the following link: <link to survey>

Should you have any questions please contact:
<Name, email, telephone >

If you experience any difficulty accessing the online system please contact:
<name, email, telephone>

Round1

Survey page 1 introductory text:

Thank you for registering to take part in the MOMENT Delphi study.

In this study we are aiming to find out which outcomes of research are important to clinicians and should be included in a core outcome set for future studies of otitis media with effusion in children with cleft palate.

We have carried out a systematic review to identify outcomes that have been measured in previous studies and also asked our study advisory group to identify any further outcomes that may have been missed. The outcomes identified are listed below. We would like you to review this list and score the importance of each outcome to you, as a clinician, when you consider this question:

What outcomes influence your management of children with cleft palate, with, or at high risk of, otitis media with effusion (OME)?

Please review the outcomes listed and score their importance in the management of children with cleft palate and otitis media with effusion on a scale of 1 to 9, with 1 being not important at all and 9 being completely critical.

Please enter a score for all outcomes. If you feel unable to comment based on your clinical experience please select 'Unable to score'.

When you are reviewing the outcomes list please remember that at this stage we are only interested in **what** should be measured. The 'how' an outcome should be measured and timing of assessments will be reviewed later, once the core outcome set has been identified.

There may be other outcomes that might be beneficial or harmful, short term or long term that you find clinically important. If you would like to add any outcomes to the list please add them in the space provided at the bottom of the page.

If at any time you wish to log out and return to your answers at a later date please use the 'Save for later' button at the bottom of the page.

Phase two survey format

Example:

MOMENT: Developing a core outcome set for children with cleft palate and otitis media with effusion.

Dear <first name>

Thank you for your responses in round one. The Delphi process is designed to develop a consensus between participants on the most important outcomes in the treatment of children with cleft palate and otitis media with effusion.

With this in mind we have summarised the outcomes from round 1 of the survey together with a reminder of your own responses. The summarised results represent the results from all ***<stakeholder group>*** who have taken part.

We would now like you to review the outcomes listed in this survey and re-score their importance. We would also like you to indicate whether or not you think each outcome should be part of the core outcome set.

We would also like to remind you that it is very important that you complete the questionnaires in each round. The reliability of the results could be compromised if people drop out of the study before it is completed because they feel that the rest of the group does not share their opinions. If people drop out because they feel their opinions are in the minority, the final results will overestimate how much the sample of participants agreed on this topic.

All responses will be anonymous. However, to help us track completion of each round please fill in your unique identifier when asked in the survey.

Your unique identifier is: XXXXXX

If you have not yet registered you will be asked to do so at the beginning of the survey and a unique identifier generated.

The survey can be accessed here: <link to survey>

If at any time you would like to discuss the Delphi survey please contact:
name,
Email
telephone>

If you experience any difficulty accessing the online system please contact:
<name, email, telephone>

Phase three survey format

Example:

MOMENT: Developing a core outcome set for children with cleft palate and otitis media with effusion.

Dear <first name>

Thank you for your responses in round two. In the previous round the results were summarised according to the clinical role of participants. In this round the results from each stakeholder group are presented to all who have taken part regardless of clinical speciality.

With this in mind we would now like you to review the outcomes listed in this survey and re-score their importance. We would also like you to indicate whether or not you think each outcome should be part of the core outcome set.

We would also like to remind you that it is very important that you complete the questionnaires in each round. The reliability of the results could be compromised if people drop out of the study before it is completed because they feel that the rest of the group does not share their opinions. If people drop out because they feel their opinions are in the minority, the final results will overestimate how much the sample of participants agreed on this topic.

All responses will be anonymous. However, to help us track completion of each round please fill in your unique identifier when asked in the survey.

Your unique identifier is: XXXXXX

The survey can be accessed here: <link to survey>

If at any time you would like to discuss the Delphi survey please contact:
name,
email,
telephone

If you experience any difficulty accessing the online system please contact:
<name, email, telephone>

All rounds reminder email

MOMENT: Developing a core outcome set for children with cleft palate and otitis media with effusion.

Dear <first name>

<round number> of the MOMENT Delphi survey to identify a core outcome set is now open. If you would like to take part please complete your response by <date>.

The survey can be found at: <link>

<For round2 and 3 only>: your unique identifier is:

Please remember that it is important for participants to complete all rounds of the survey.

If you have previously registered and saved your scores for later please remember to log in and submit your responses.

Thank you for your help to develop a core outcome set for trials of otitis media with effusion in children with cleft palate.

If at any time you would like to discuss the Delphi survey please contact:
name,
email,
telephone

If you experience any difficulty accessing the online system please contact:
<name, email, telephone>

Welcome registration email

Email Title: *MOMENT- Developing a core outcome set for children with cleft palate and otitis media with effusion.*

Thank you for registering to take part in the MOMENT study Delphi to develop a core outcome set for children with cleft palate and otitis media with effusion.

You have successfully registered for the study and your unique identifier is : MOXXXXXX. Please keep this safe.

The online Delphi can be accessed here: Delphi Survey

If at any time you would like to discuss the Delphi survey please contact:
Name
Email:
Telephone:

If you have not registered for this study please contact us at the email address below so we can investigate why the email has been generated.

If you experience any difficulty accessing the online system please contact:<name, email, telephone>
