# Supplementary material for: MOMENT – Management of Otitis Media with Effusion in Cleft Palate: protocol for a systematic review of the literature and identification of a core outcome set using a Delphi survey
Source: Trials. 2013 Mar 12;14:70. doi: 10.1186/1745-6215-14-70 (PMC3716725; doi:10.1186/1745-6215-14-70)
Supplement: Additional file 4 — Online system screenshots. [file 1745-6215-14-70-S4.pdf]

# Additional Document 4 -Online System Screenshots

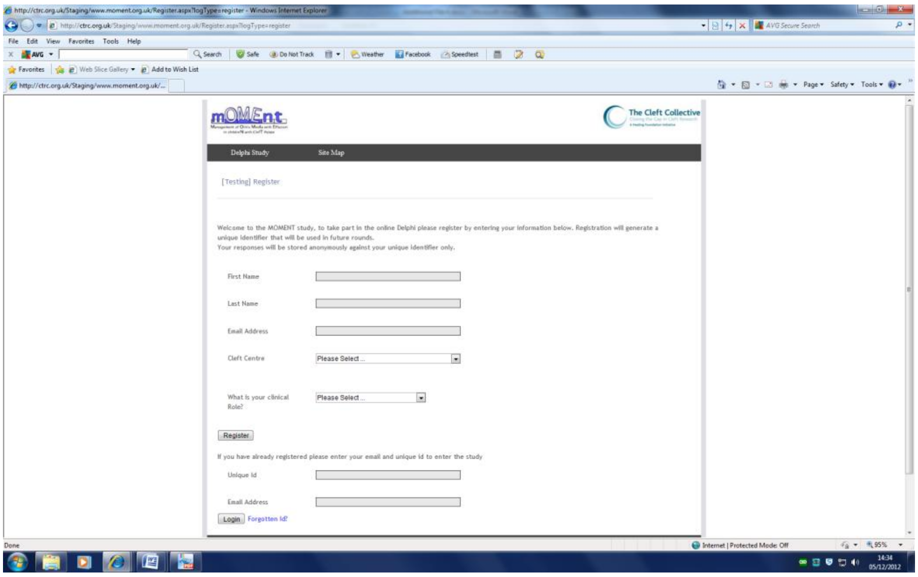


Registration

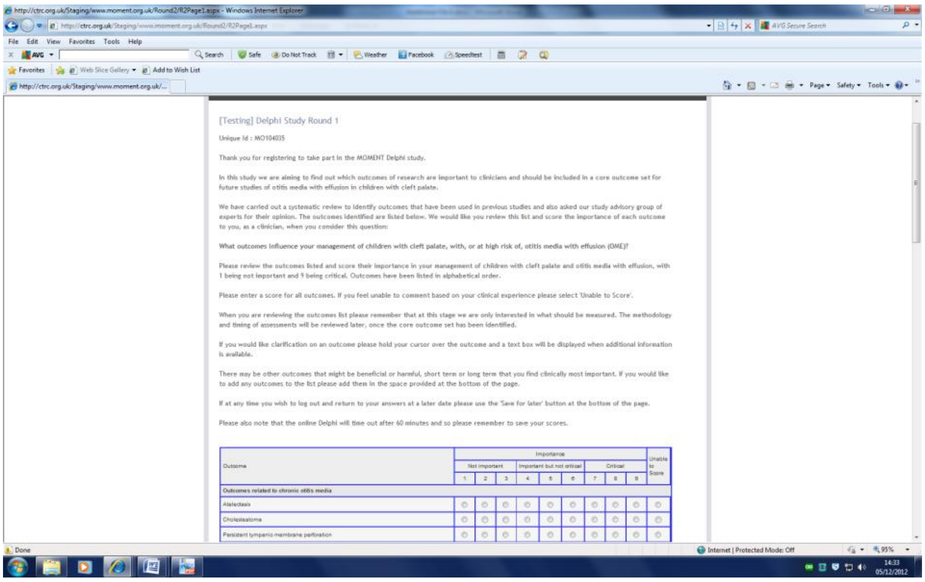


Example of outcomes list

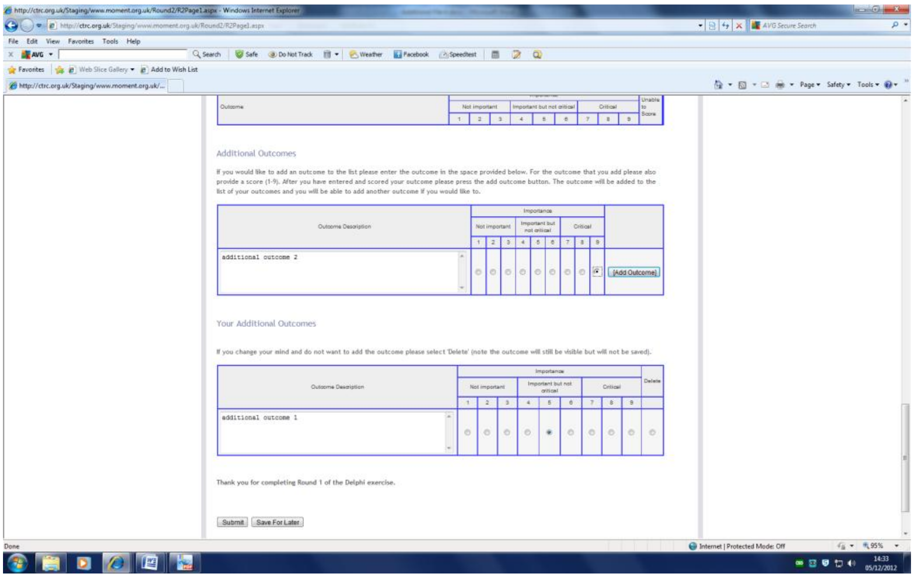


Example of add additional outcomes function
